# Fibroid expulsion: a unique presentation of mechanical small bowel obstruction 11 years after uterine artery embolization: a case report

**DOI:** 10.1186/s13256-021-02917-z

**Published:** 2021-07-09

**Authors:** Jeffrey L. Roberson, Lauren N. Krumeich, Nabil F. Darwich, Victor Babatunde, Dorottya Laczko, Andrew Albee, Zhaohai Yang, Amr El Jack, Richard Shlansky-Goldberg, Mary DeAgostino-Kelly, Benjamin M. Braslow

**Affiliations:** 1 Department of Surgery, Hospital of the University of Pennsylvania, 3400 Spruce Street, 4 Maloney, Philadelphia, PA 19104 USA; 2 Perelman School of Medicine, University of Pennsylvania, Philadelphia, USA; 3 Department of Radiology, Hospital of the University of Pennsylvania, Philadelphia, USA; 4 Department of Pathology & Laboratory Medicine, Hospital of the University of Pennsylvania, Philadelphia, USA; 5 Department of Obstetrics and Gynecology, Hospital of the University of Pennsylvania, Philadelphia, USA; 6 Division of Trauma, Surgical Critical Care, and Emergency Surgery, Hospital of the University of Pennsylvania, Philadelphia, USA

**Keywords:** Uteroenteric fistula, Fibroid, Small bowel obstruction, Uterine artery embolization, Case report

## Abstract

**Background:**

Uterine artery embolization in the treatment of uterine leiomyoma has been rarely associated with dislodgement and expulsion of infarcted uterine fibroids through the vagina, peritoneum, or bowel wall, predominantly occurring within 6 months of uterine artery embolization.

**Case presentation:**

We present the case of a 54-year-old African American woman who underwent uterine artery embolization 11 years prior and developed mechanical small bowel obstruction from the migration of fibroid through a uteroenteric fistula with ultimate impaction within the distal small bowel lumen. Small bowel resection and hysterectomy were curative.

**Conclusions:**

Uteroenteric fistula with small bowel obstruction due to fibroid expulsion may present as a delayed finding after uterine artery embolization and requires heightened awareness.

## Background

Leiomyomas, or fibroids, are benign tumors of the smooth muscle that originate in the myometrium. It is estimated that, by age 50 years, 70% of women will have at least one leiomyoma, and 30% of those women will be symptomatic [1, 2]. Symptoms may include abnormal uterine bleeding, abdominal pain, dysmenorrhea, and obstructive symptoms due to compression on the bladder or rectum [3]. Uterine artery embolization (UAE) is an effective, noninvasive treatment modality for symptomatic fibroids [4]. While typically well tolerated, the American College of Obstetricians and Gynecologists found that the vast majority (87.5%) of complications from UAE occur within 3 months of the procedure [5] and can include prolapse and transvaginal expulsion of a devascularized fibroid, affecting 1–10% of patients [6, 7]. Rarely described fistulae to the bladder and bowel lumen have been documented in the literature between 21 days and 6 months post embolization [8–11], thought to be related to uterine necrosis from devascularization. This is the first known case report of delayed development of a uteroenteric fistula with expulsion of a fibroid causing a small bowel obstruction (SBO) more than a decade after UAE. Providers must maintain awareness of the delayed presentation of this surgical emergency that may become increasingly prevalent as time accumulates from the introduction of UAE in 1995 [12].

## Case presentation

A 54-year-old gravida 1 para 1 African American woman with a history of hypertension, uncomplicated diverticulitis, and multiple uterine fibroids status post UAE presented to the emergency department (ED) with 3 days of severe, colicky abdominal pain, inability to tolerate oral intake, and bilious emesis.

The patient’s uterine fibroids were originally diagnosed in the setting of heavy menstrual bleeding and intermenstrual bleeding. Prior to intervention, her uterus measured 10.8 × 11.3 × 17 cm and exhibited three predominant, viable fibroids, the largest of which was 6 × 6 × 6 cm. She underwent UAE with polyvinyl alcohol in 2009. Magnetic resonance imaging (MRI) of the pelvis the day following her UAE demonstrated appropriate infarction of all fibroids. Three months following the UAE, her uterus had decreased in size, her fibroids appeared necrotic, and she reported complete cessation of her intermenstrual bleeding as well as lighter, more regular menstruation. Ten years following UAE, her intermenstrual bleeding recurred, and MRI suggested the presence of one viable subserosal leiomyoma. She was offered hysterectomy but declined because of personal preference. In the subsequent year prior to presentation, she developed drainage from the vagina consistent with enteric contents but declined further workup.

**Fig. 1:**
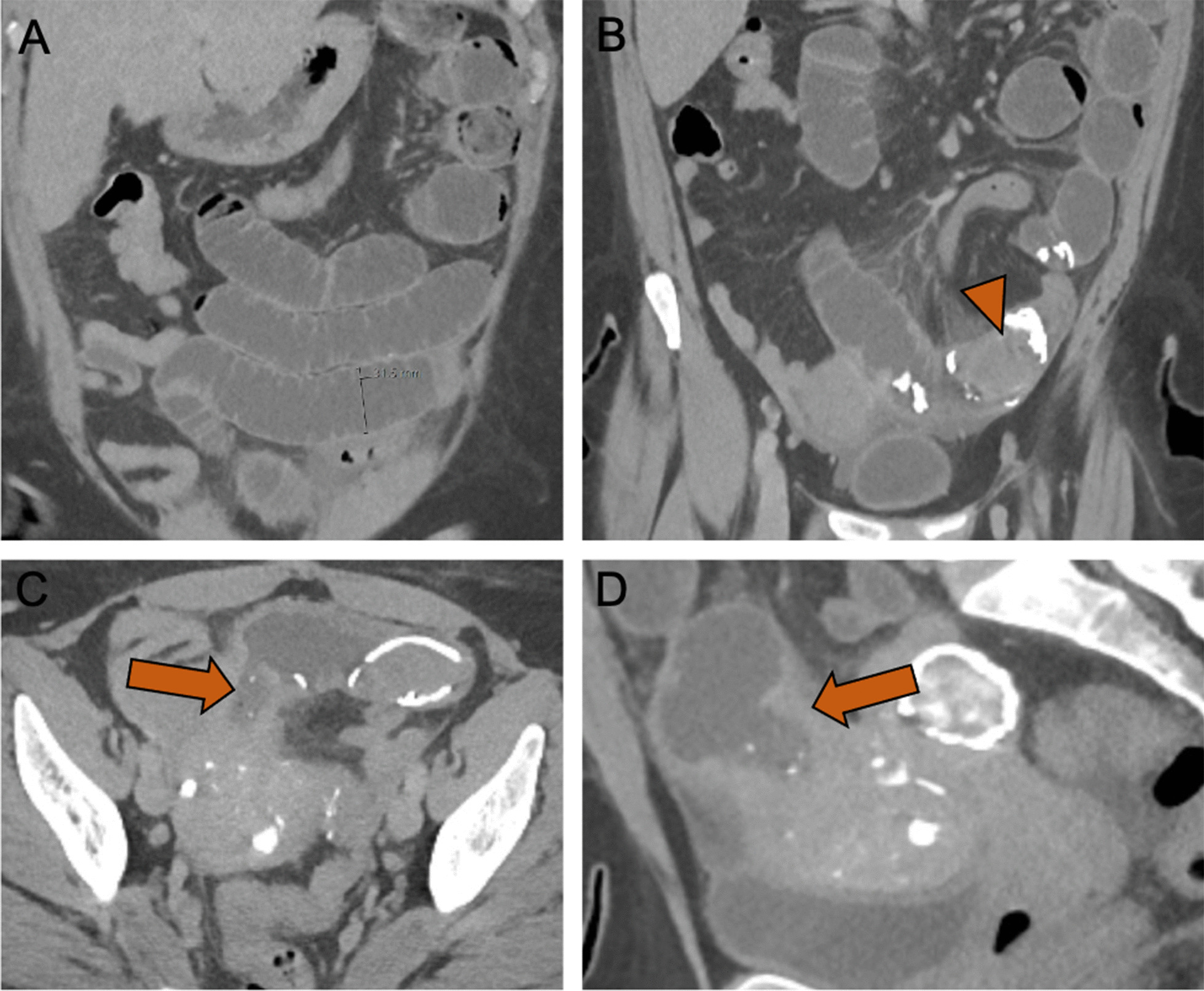
Contrast-enhanced CT images with coronal (**A**, **B**), axial (**C**), and sagittal (**D**) images. **A** Small bowel obstruction with dilated small bowel loop measuring up to 3.2 cm. **B** Small bowel obstruction secondary to an obstructing uterine leiomyoma in the ileal lumen (arrowhead). **C**, **D** Migration of leiomyoma into the ileal lumen due to an uteroenteric fistula between the uterine fundus and the ileum (arrows)

**Fig. 2:**
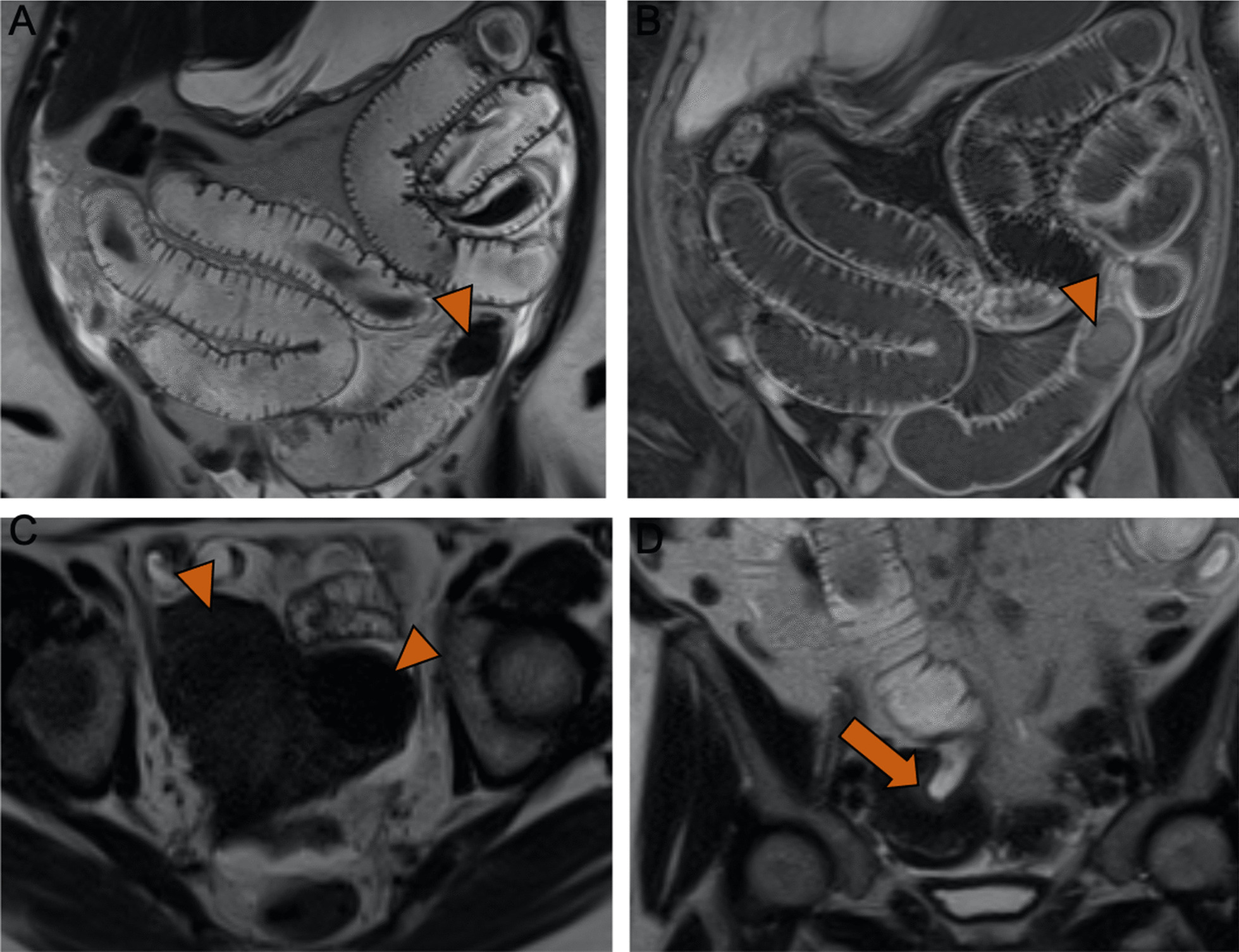
Magnetic resonance imaging with coronal T2-weighted (**A**, **C**), axial T2-weighted (**B**), and coronal contrast-enhanced T1-weighted (**D**) images. **A** Small bowel obstruction is again noted with an intraluminal T2-dark lesion within the ileum (arrowhead). **B** Similar T2-dark lesions (arrowheads) are noted in the uterus, in keeping with uterine leiomyomas. **C**, **D** A uteroenteric fistula (arrow); the uterine fundus and the ileum served as a conduit for migration of a leiomyoma into the ileum, leading to the small bowel obstruction

On presentation to the ED, the patient was not obstipated. She was diffusely tender to palpation without rebound or guarding, leukocytosis, acute kidney injury, or lactic acidosis. Her abdominal surgical history included laparoscopic cholecystectomy and cesarean section. She had an unremarkable colonoscopy within the year prior to presentation. She underwent computed tomography (CT) scan of the abdomen and pelvis with intravenous contrast, which demonstrated an SBO from an obstructing intraluminal fibroid that had migrated through a uteroenteric fistula (Figs. 1 and 2). Additionally, a pelvic MRI with gadolinium was obtained to assess for malignancy and demonstrated persistent viability of a 3.7 × 3.5 × 3.8 cm subserosal fibroid. There was no imaging evidence of leiomyosarcoma.

**Fig. 3:**
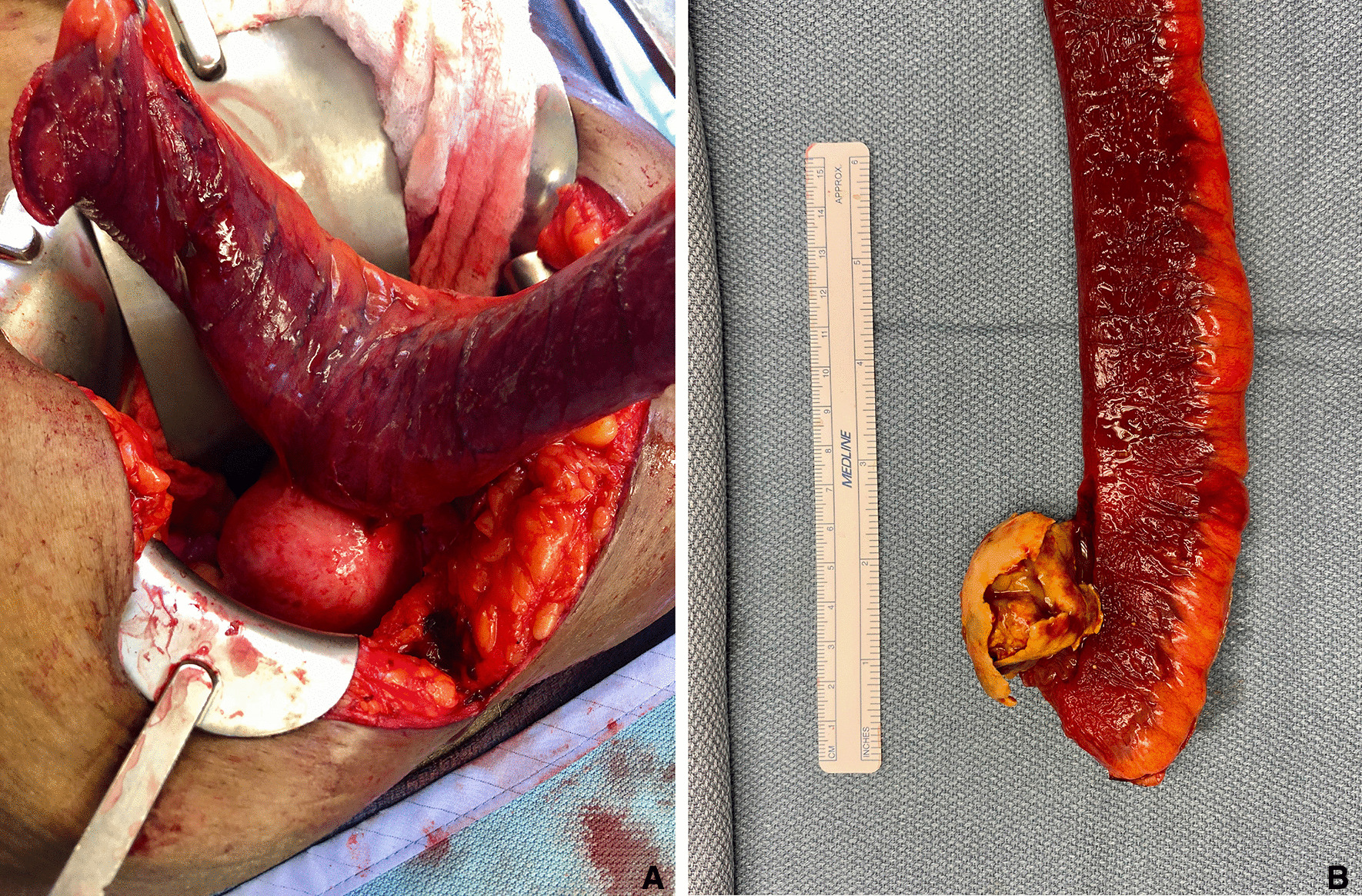
**A** Resected small bowel adherent to uterus through uteroenteric fistula. **B** Postprocedural enterotomy reveals the fibroid that had migrated into distal small bowel and caused small bowel obstruction

Following 36 hours of nasogastric decompression and intravenous hydration, she underwent lower midline laparotomy that revealed a broad-based fistula between the distal small bowel and uterus (Fig. 3A). An obstructing calcified mass was palpated intraluminally proximal to the ileocecal valve and was resected within a 35-cm specimen of small bowel en bloc with the uterus (Fig. 3B). Another uterine fibroid was adherent to sigmoid colon mesentery and was removed with sharp dissection without injury to the sigmoid colon or its mesentery. A primary side-to-side small bowel anastomosis was created. She was extubated in the operating room with a nasogastric tube remaining in place to allow for ongoing decompression prior to return of bowel function.

Postoperatively, she had prerenal acute kidney injury (creatinine maximum 2.2 mg/dL) that was fluid-responsive and resolved within 3 days. She had complete return of bowel function by postoperative day 5 and was subsequently discharged home on a regular diet. She was seen at 1- and 2-month intervals following her operation and was found to be tolerating her preoperative diet and having regular bowel function with cessation of her transvaginal enteric discharge.

**Fig. 4:**
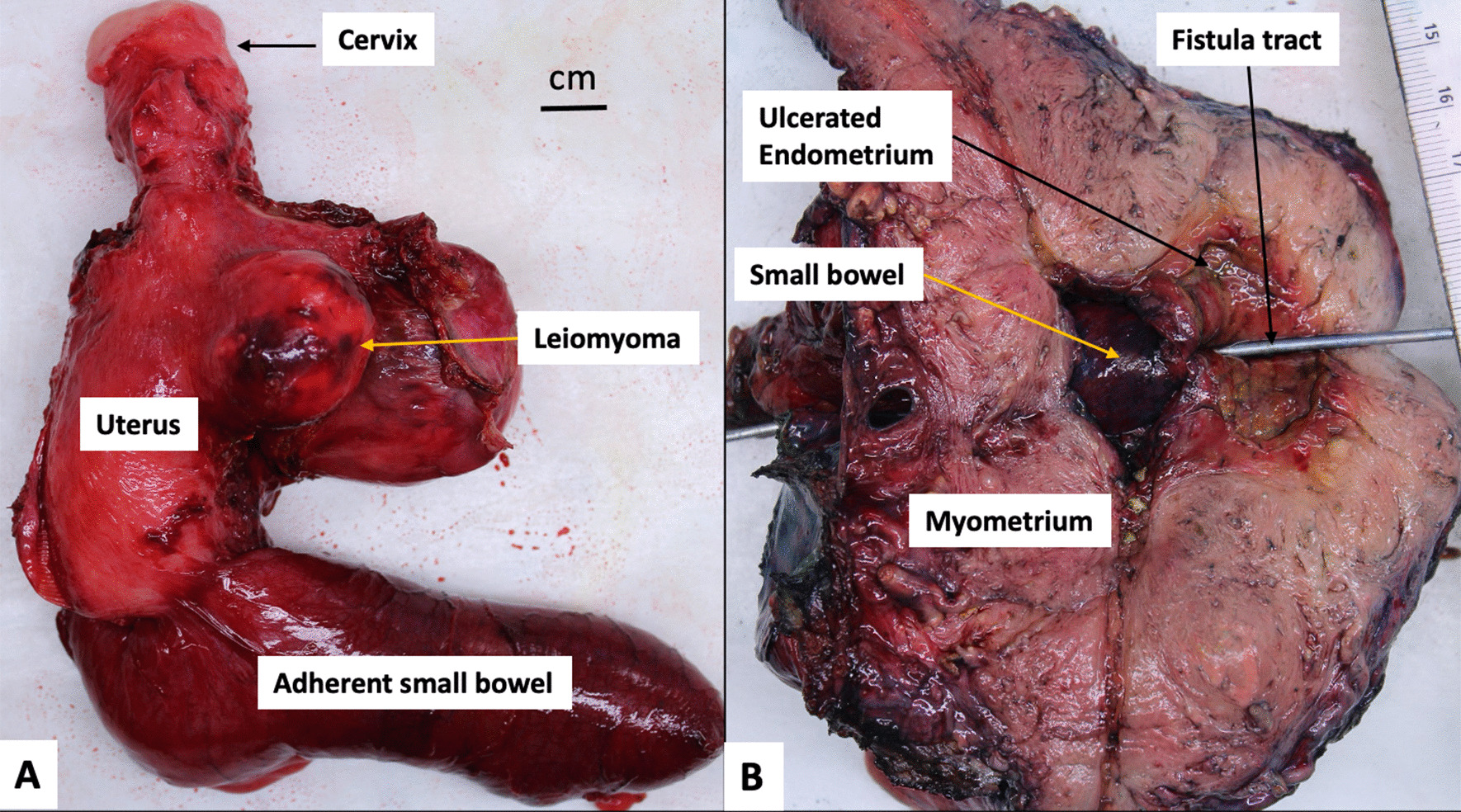
Gross specimen of resected uterus with adherent small bowel. **A** Overall specimen showing adherent small bowel and uterus with leiomyoma. **B** Cut surface showing the adherent small bowel with uteroenteric fistula (probe), which is grossly ulcerated

**Fig. 5:**
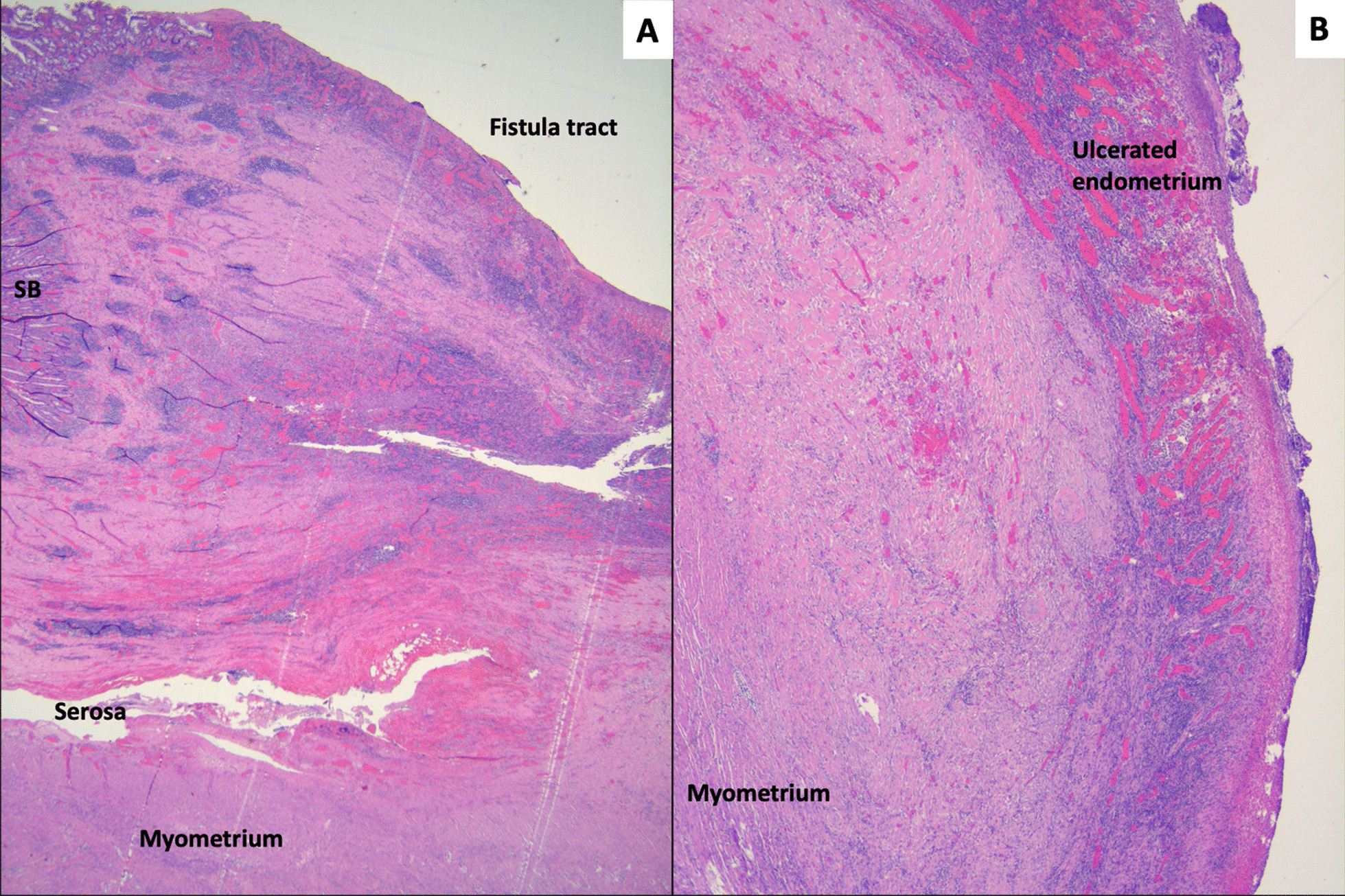
Microscopic images of the uteroenteric fistula. **A** The fistula tract showing continuation between small bowel mucosa (SB, upper left) and ulcerated endometrium. Also note the adherent small bowel and uterine serosa (original magnification ×12.5). **B** Ulcerated endometrium and myometrium (original magnification ×25).

Gross pathology of the intraluminal mass revealed a 4.9 cm hyalinized leiomyoma with calcified shell. The small bowel was found to have edema, vascular congestion, and focal hemorrhage, consistent with ischemic changes secondary to small bowel obstruction. On gross examination, the uterus demonstrated a leiomyoma and adhesion to the small bowel, which contained the uteroenteric fistula (Fig. 4). Microscopically, the fistula tract showed continuation between small bowel mucosa and ulcerated endometrium with granulation tissue (Fig. 5).

## Discussion and conclusions

Uteroenteric fistula leading to enteric impaction from an expelled fibroid is a rare cause of SBO. More commonly, an SBO in the setting of fibroid disease is secondary to external compression of the small bowel from a large residual leiomyoma [13] or adhesion formation after UAE [14].

Uteroenteric fistula formation has previously been documented only in the subacute postprocedural period after UAE [15]. This is the first case to describe a uteroenteric fistula with only a distant history of UAE. Due to the patient’s preference to avoid any workup when she first developed transvaginal enteric drainage, the natural progression of this fistula was able to be observed, with an SBO developing approximately 1 year after she first developed transvaginal enteric drainage suggestive of a fistula. For any patient with a history of UAE or persistent fibroid disease who develops transvaginal enteric drainage, we recommend obtaining an MRI to investigate the presence of a fistula and allow for intervention before progression to an SBO.

While the remote ischemic insult of UAE may have predisposed this patient to develop a uteroenteric fistula approximately 10 years after her procedure, it is also conceivable that her recurrent viable leiomyomas led to pressure necrosis in the small bowel wall. Although this has never before been described for uteroenteric fistula, this mechanism is similar to that of a cholecystoenteric fistula, which predisposes to gallstone impaction in the lumen of the small bowel. This mechanism is suggested by the ischemia identified in her small bowel and by the fact that the patient was found in the operating room to have a second fibroid adherent to the sigmoid mesentery, which may have been the early stages of another developing uterocolonic fistula.

The primary goal of surgical management is to relieve the small bowel obstruction. For gallstone ileus, if the obstructed small bowel appears viable, enterotomy in healthy-appearing bowel is often utilized, allowing the obstructing stone to be milked retrograde toward the enterotomy [16]. The typical size of the gallstone leading to this obstructive phenomenon is 4.14 cm, similar in size to the obstructing leiomyoma in this case [17]. Thus, an enterotomy for fibroid extraction would have been reasonable to consider. Based on the degree of edema, vascular congestion, and focal hemorrhage in the small bowel identified on pathology, we would suggest a similar management strategy with proximal enterotomy to avoid an incision within compromised small bowel. However, considering the calcified nature of the fibroid as well as its tendency to fracture when manipulated, we would exercise extreme caution if employing this strategy to avoid small bowel luminal damage and/or only partial extraction of the fibroid. If there is concern that the small bowel is ischemic at the site of the obstruction, that the small bowel lumen will be compromised by milking the fibroid, or that contents of the fibroid will be lost with manipulation of the fibroid and lead to distal obstruction, then a small bowel resection should be employed.

The secondary surgical goal is to remove the fistula to prevent further expulsion of fibroids. Dissimilar to a gallstone ileus, in which the fistula is often in the duodenum and the obstruction at the ileocecal valve, the site of the obstruction in this case was in close proximity to the fistula. As a small bowel resection was required to address the broad-based fistula and prevent small bowel narrowing, we employed a single en bloc small bowel resection, fistulectomy, and hysterectomy to manage the fistula and the obstruction concurrently. Had the small bowel obstruction been significantly distal to the fistula, we would have managed the small bowel obstruction separately with either a second small bowel resection or an enterotomy with fibroid extraction after considering the aforementioned factors.

In summary, uteroenteric fistula with an impacted intraluminal fibroid is a rare cause of SBO that has never before been described without recent exposure to UAE. In this case, an en bloc resection was performed with the uterus, small bowel, fistula, and obstructing fibroid, allowing for a single small bowel anastomosis without further damage to the small bowel or fracturing of the fragile fibroid. Despite similarities to gallstone ileus, uteroenteric fistulae have unique characteristics based on the location of the fistula and the nature of the fibroid that require careful consideration prior to employing a surgical management strategy.

## Data Availability

Data sharing is not applicable to this article as no datasets were generated or analyzed during the current study.

## Abbreviations

CT: Computed tomography
ED: Emergency department
IV: Intravenous
MRI: Magnetic resonance imaging
UAE: Uterine artery embolization
SBO: Small bowel obstruction
